# Sorption of Nanomaterials to Sandstone Rock

**DOI:** 10.3390/nano12020200

**Published:** 2022-01-07

**Authors:** Christian Scheurer, Rafael E. Hincapie, Elisabeth Neubauer, Astrid Metz, Daniel Ness

**Affiliations:** 1 DPE Department Petroleum Engineering, Montanuniversität Leoben, Franz-Josef-Straße 18, 8700 Leoben, Austria; scheurerchristian@me.com; 2 OMV Upstream Technology & Innovation, TECH Center & Lab, OMV Exploration & Production GmbH, 1020 Vienna, Austria; Elisabeth.Neubauer@omv.com (E.N.); Astrid.Metz@omv.com (A.M.); 3 Evonik Operations GmbH, Research, Development & Innovation, D-63450 Hanau, Germany; daniel.ness@evonik.com

**Keywords:** silica nanostructured particles, nanoparticle adsorption, batch sorption, rock-fluid interactions, single phase core-flood, sandstone, fluid-fluid interactions

## Abstract

We investigated the interaction of silica nanostructured particles and sandstone rock using various experimental approaches, such as fluid compatibility, batch sorption and single-phase core-floods. Diol and polyethylenglycol (PEG) surface-modified nanostructured silica materials were tested using two brines differing in ionic strength and with the addition of sodium carbonate (Na_2_CO_3_). Berea and Keuper outcrop materials (core plug and crushed samples) were used. Core-flood effluents were analysed to define changes in concentration and a rock’s retention compared to a tracer. Field Flow Fractionation (FFF) and Dynamic Light Scattering (DLS) were performed to investigate changes in the effluent’s size distribution. Adsorption was evaluated using UV–visible spectroscopy and scanning electron microscopy (SEM). The highest adsorption was observed in brine with high ionic strength, whereas the use of alkali reduced the adsorption. The crushed material from Berea rock showed slightly higher adsorption compared to Keuper rock, whereas temperature had a minor effect on adsorption behaviour. In core-flood experiments, no effects on permeability have been observed. The used particles showed a delayed breakthrough compared to the tracer, and bigger particles passed the rock core faster. Nanoparticle recovery was significantly lower for PEG-modified nanomaterials in Berea compared to diol-modified nanomaterials, suggesting high adsorption. SEM images indicate that adsorption spots are defined via surface roughness rather than mineral type. Despite an excess of nanomaterials in the porous medium, monolayer adsorption was the prevailing type observed.

## 1. Introduction

Nanotechnology has gained interest over the last decade, with beneficial applications in upstream and downstream. For the former, nanotechnology provides new and promising applications when applied in reservoir areas, for example, in enhanced oil recovery (EOR) or drilling-related activities. For instance, to reduce formation damage during drilling, as well as to enhance production in mature fields [1,2,3], protect the reservoir formation, reduce fluid loss and prevent shale swelling [4,5,6,7,8,9]. Due to their small size, nanomaterials have the ability to pass through reservoir rock and can be surface active, which is a key requirement for influencing oil–rock–water interfaces.

The oil industry has mainly focused on silica nanomaterials (SiO_2_) for economic reasons. However, research includes studies in many other developed nanomaterials. Alomair et al. [10], for instance, described the use of SiO_2_, NiO, TiO_2_ and Al_2_O_3_ particles in EOR. One advantage is that their diameters are smaller than the pore throats of thief zones, thus improving the flooding potential without reducing permeability. This effect is achieved by an increased area of fluid contact by the driving fluid and an increased microscopic sweep efficiency [11,12]. In EOR, pore throat plugging can be a desired effect: since nanomaterials can be adsorbed on the grain walls of pore throats, they increase a bulk pressure difference by narrowing the pore channel. This leads to an increase in drive fluid velocity and higher pressures, forcing trapped oil drops into the flow [11,13].

Much research has been presented on the application of nanomaterials to recovery oil, including its description and usage [14,15,16,17,18,19]. Various mechanisms are considered when explaining nanomaterial effects, such as wettability alteration due to disjoining pressure [1,20], spontaneous emulsion formation and emulsion stabilisation [17,18], change of interfacial tension (IFT) between reservoir fluids and change of flow properties of the porous medium [1,14,15,16,17,18,19,20]. Among other mechanisms taking place, nanoparticle adsorption is a very important topic in nanomaterial application. The topic and other unique properties of the nanomaterials has been broadly covered by researchers, e.g., Petosa et al. [21] and Zhang et al. [22]. The possible adsorption of the nanoparticles in the porous media has been studied in relation to wettability and recovery [21,22]. Nanoparticle adsorption is controlled by multiple factors. Li et al. [23] observed, by the mean of advanced surface wetting visualisation, an increase in NP adsorption onto Berea sandstone when the NP concentration rose. The pH of the nanofluids also had an impact on the adsorption process, with a low pH (pH = 2.01) favouring uniform NP adsorption. As the nanofluids’ pH increased to 4.84, a thicker layer of NPs adsorbed onto the rock.

Nanomaterials, for instance, can lead to the formation of clusters during their application in porous media. Understanding the formation of clusters is important because they can be retained within the pore throats. With their large surface area, nanomaterials tend to agglomerate quickly if they are not stabilised since this minimises their surface energy. According to Huh et al. [24], nanomaterials in dispersion are subject to Brownian motion and therefore collide with each other. When a collision occurs, depending on the magnitude of attraction and repulsion forces, the particles will form clusters or stay dispersed. Electrostatic stabilisation is achieved by the particle’s repelling forces caused by their Zeta potential. Zeta potential is the electrical potential measured at the slipping plane of a suspended particle under an electrical field [25]. This generated repulsion is weakened in the presence of dissolved salts in brines: electrolytes destabilise the particle dispersion by compressing the electric double layer (EDL). An increase in electrolyte concentration, therefore, reduces the energy barrier. The kinetic energy of the particles then surface charges dictate the probability of aggregation [26].

Nanomaterials interact with the surrounding fluid, other nanomaterials and the rock surface in a porous media. These interactions are governed by static interaction, thermodynamic forces and hydrodynamic forces. Upon collision with the rock, particles tend to stay in the stagnant points of the flow surface. The DLVO theory (Derjaguin, Landau, Verwey and Overbeek) is used to predict van der Waals (vdW) attraction and EDL repulsion in this case. Zhang et al. [22] showed the interaction energy of nanomaterials with Boise sandstone, where vdW attraction is dominant. Electrostatic repulsion greatly depends on the surface potential and the ionic strength of the brine. Strong repulsion only exists in low-salinity brine (10 mM/0.051 wt% NaCl). In most injection brines, the salinity and therefore the ionic strength is much higher, and therefore, the double layer repulsion might be negligible. Steric stabilisation—polymer coating the outside of particles—can provide enough repulsion to stabilise the dispersion in this case [22].

According to Lauth and Kowalczyk [27], Irving Langmuir’s model is one of the most used to describe particle adsorption. It assumes that the surface of the adsorbent has a limited number of equal spots where particles can be bound. The probability of adsorption to a certain spot does not depend on the occupancy of neighbouring spots. If nanomaterials are injected into a fresh sample of rock, the thermodynamic force attracts nanomaterials to the rock surface. After continuous injection, at some point, equilibrium between nanomaterials in the dispersion and on the surface will be reached. The subsequent post-flush does not contain nanomaterials, and the thermodynamic force is reversed. Desorption occurs and removes nanomaterials from the surface. The surface properties of the rock grains and vdW attraction are, therefore, the main contributors to nanoparticle adsorption. Therefore, the size of nanomaterials, surface coating and rock lithology are defining factors [22].

## 2. Overall Aim

In this work, the sorption of surface-modified silica nanomaterials to reservoir rock is evaluated. Multiple variables are deemed highly important, such as rock mineralogy, nanomaterial surface charge and reservoir brine composition. Two sandstone outcrop rocks were considered, namely Keuper and Berea. Two surface-modified silicon dioxide particle samples with different surface modifications and reservoir brine to account for the effect of divalent cations were used. Further, various methods were developed to characterise the nanomaterial’s effects. Overall, with this work, we cover different topics:

1. Assessing rock sample characterisation by means of routine core analysis, particle size distribution, Brunauer–Emett–Teller (BET) and Zeta potential experiments;
2. Evaluating the fluid–fluid (brine–nanomaterial) interaction using a compatibility test;
3. Evaluating nanomaterial and rock interaction using static batch sorption experiments and ultraviolet–visible (UV–Vis) spectroscopy;
4. Define the effect of the nanomaterials on the reservoir rock through single-phase core floods and size and concentration analysis of the effluents.

This work provides additional information to the literature body by combining data from multiple sources (to clearly determine the conditions and resulting behaviour), which, to the best of the authors’ knowledge, have not been fully addressed. Furthermore, supporting the observations in core floods and batch sorption’s with various sources of effluent analysis and scanning electron microscopy (SEM) provide researchers with a valuable set of data.

Experiments consisted of batch sorption experiments and single-phase core flood experiments. In the batch sorption experiment, crushed rock samples were mixed with various solutions of nanofluids in brine. The supernatant was investigated for a change of nanomaterial concentration, providing indications, such as which rock type, brine and temperature will support/prevent adsorption.

Sorption was evaluated using UV–visible spectrophotometric absorption for nanoparticle concentrations in the batch sorption experiments and effluents of the core flood experiments. Porosity and permeability were measured before and after core flooding in order to investigate pore plugging. Scanning electron microscopy (SEM) was used to analyse which minerals the nanomaterials preferentially attached to and to look at plugging effects (visualise adsorbed nanomaterials). Flow Field Flow Fractionation (FFF) was used to investigate the size distribution of nanomaterials before and after core floods. In the core flood effluents, FFF was used to evaluate shifts in size distribution after interaction with the rock. Surface charges of the particles and rocks, by means of Zeta potential, were used to analyse the particle–rock interaction. Note that the simulation and derivation of mathematical models to describe the particle’s adsorption was considered out of the scope of this work.

Note that, in this work, we differentiate adsorption and absorption. We consider adsorption as the adhesion of nanoparticles to the soil’s surface, while absorption refers to the penetration of the nanoparticles into a soil matrix [8,12,21]. We deal here with rather small pores, opposite to the large pores that allow capillary forces to draw in fluids, which, in turn, permit adsorption forces to be predominant and to fill the macroscopic voids. Hence, internal surface areas play a key role in the evaluation.

As a way of delimitation, note that fluid–rock interactions in the presence of hydrocarbons were not considered as part of the scope of this work since the particular evaluations and effects we considered and presented in some other works of the authors, such as Saleh et al. [1] and Neubauer et al. [18].

## 3. Materials and Methods

### 3.1. Fluids and Rock Material

#### 3.1.1. Core Plugs

Outcrop rock from Berea and Keuper sandstone was used, and its properties are shown in Table 1. For the batch adsorption experiments, rock crushed to a coarse powder was utilised. Samples consisted of a homogeneous material of mixed grain sizes to ensure fine clay material was included as well. Core plugs were cut dry to avoid clay swelling.

**Figure 1:**
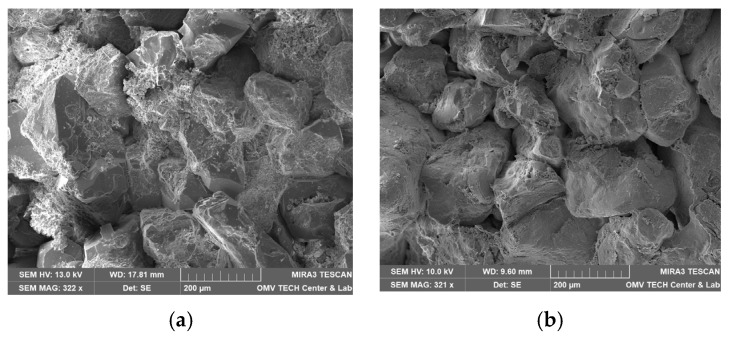
Scanning electron microscope (SEM) image measured for the core material used in this work; Berea is shown in (**a**) and Keuper in (**b**). In (**a**), quartz cement can be identified by its smooth surfaces compared to the sand grains. Kaolinite is placed between sand grains in its typical book shape. In (**b**), the sand grains are more rounded compared to Berea (**a**), but a reduced amount of clay and quartz cement is visible.

Berea sandstone is a well-sorted yellowish sandstone with approximately 87% quartz, 5% feldspar and 7% clay. The roundness is angular to sub-angular. A scanning electron microscope (SEM) image of a thin section analysis from the same outcrop is shown in Figure 1a. Pore walls are covered with feldspar or clay [1]. According to the obtained XRD data, the clay is a mix of 92% kaolinite, 7.5% chlorite and 0.7% illite by mass. Keuper sandstone is a fine-grained red-brown sandstone with dark spots. The mineralogical composition is approximately 95% quartz, 1% feldspar and 4% mica. It is well sorted and mainly grain-supported. Limonite is the iron oxide that causes the reddish colour [1,28]. A scanning electron microscope (SEM) image of a thin section analysis from the same outcrop is shown in Figure 1b.

Pore size distribution was observed to have a tendency of 8–16 µm pore throats for Berea and Keuper. Note that for the evaluations presented here, the impact of pore size distribution was not evaluated since it was regarded as not relevant. Instead, BET-specific surface area measurements were used as they revealed more insight into the adsorption process.

#### 3.1.2. Synthetic Brines

A softened injection brine, test water (TW) and a synthetic formation brine, here named formation water (FW), were selected to investigate the effect of divalent cations. Their composition and properties are shown in Table 2.

#### 3.1.3. Alkali Solution

The prepared alkali solution was 3000 ppm Na_2_CO_3_ in TW, hereafter named alkali solution (AS). This concentration was selected since it resulted in the highest emulsion stability and interfacial tension (IFT) reduction [17]. The used concentration reported by Neubauer et al. [17] was 0.1 wt% nanoparticles. First, a 50,000-ppm mother solution was prepared and subsequently diluted to create a 3000 ppm concentration when mixed with the respective nanofluid. With a pH of 9.89, 3000 ppm Na_2_CO_3_ in TW was used as a background probe for the UV–Vis measurements.

#### 3.1.4. Nanofluids

**Figure 2:**
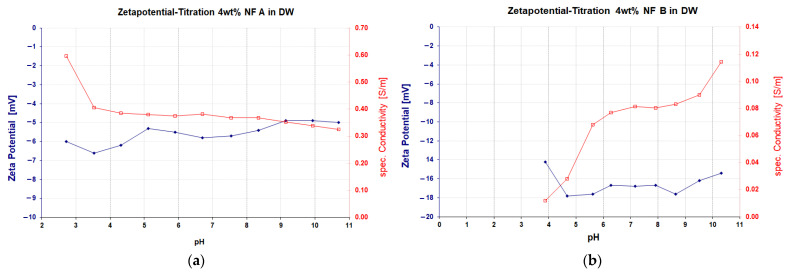
Zeta (ζ) potential and specific conductivity for the materials used in this work. Measurement was performed using deionised water as dispersion media with HCL as acid and KOH as base. Nanofluid A is shown in (**a**) and Nanofluid B in (**b**). The particles are moved relative to their diffuse electrical double layer (EDL) to form fluctuating dipoles. An alternating current, i.e., the colloidal vibration current, is generated, which subsequently allows the calculation of Zeta potential. NF B is more negatively charged due to the polar functionality of its surface coating. This should result in better colloidal stability. One could argue that no marked variation can be considered as a function of pH since the Zeta potential appears almost constant (measured error +/−5 mV).

**Figure 3:**
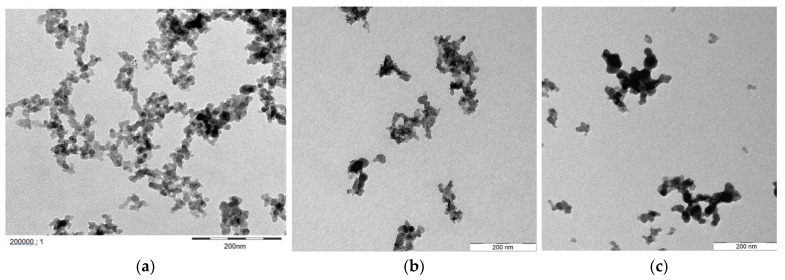
Transmission Electron Microscope (TEM) images measured for the nanomaterials used in this work. Unmodified silica nanomaterials shown in (**a**), Nanofluid A in (**b**) and Nanofluid B in (**c**).

The used nanomaterials were provided by *Evonik Operations GmbH* (Hanau, Germany) in the form of dispersions of fumed silica particles. The dispersion does not have a high salt content but might have silanes in excess. These could react and hydrolyse while drying, which might explain the unexpected shapes seen in previously conducted SEM images. The nanofluids differ in the surface modification applied to them and are hereafter called NF A and NF B. NF A contained PEG chains as surface modifications and was rather unreactive during in-house corrosion tests. NF B had two 2-diol groups grafted to the surface of the particles and showed higher corrosive potential. The base material of both is fumed amorphous silica (AEROSIL^®^ fumed silica, Evonik Hanau—Germany). The surface of nanomaterial A is coated with polyethylene glycol (PEG), while the surface of nanomaterial B has a diol functionality. ζ potential (Figure 2) was assessed using a Nano Z manufactured by Malvern Zetasizer Pro (Malvern Panalytical Ltd., Malvern, UK), which relies on using the dynamic light scattering (DLS) technique. Their properties can be seen in Table 3 Measurements at room temperature were conducted at 22 °C. The Transmission Electron Microscope (TEM) images (Jeol 2010F, 200 KV, Tokyo, Japan) shown in Figure 3. depict that the modified particles show loose aggregates, which can easily break apart.

To evaluate the fluid–fluid and fluid–rock interactions, NF A and NF B were diluted in two concentrations, each in TW and FW. The mixtures containing the alkali solution were mixed in 0.1 wt% only. A mixture of FW and Na_2_CO_3_ was not used due to incompatibility found in earlier experiments resulting in calcium carbonate precipitations.

#### 3.1.5. Tracer

Ammonium Thiocyanate (NH_4_SCN) provided by VWR International (Vienna, Austria) was selected as the chemical tracer for the core floods. Pre-emptive spectrophotometry tests showed the influence of the tracer below 260 nm. Therefore, the selected wavelength for all interpretations of the UV–Vis spectrophotometer data was 270 nm. To create a tracer concentration of 30 ppm in the nanofluid slugs, 1000 ppm NH_4_SCN solution was added to the brines. The effluent samples were eventually diluted to 1:3 and analysed using Ion Chromatography (IC).

### 3.2. Batch Sorption Experiments

This experiment was used to determine the interaction of rock material with nanomaterials and brines in various combinations and conditions. Batch sorption experiments were conducted to quantify the maximum mass of absorbable nanomaterials for a given combination of rock–fluid. Therefore, crushed rock material was mixed with nanofluids and alkali solution, tracking the concentration of nanomaterials using UV–Vis spectrophotometry. Hence, 5 g of both types of crushed rock material was mixed with 20 mL of each respective solution. Two samples of each combination were placed in a sample holder inside a heating cabinet at room temperature (RT) and 60 °C. The sample holder was rotating the samples for 24 h at approximately 35 rpm. Subsequently, samples were left resting for one hour for gravity settling. Then, the suspension was filtered with 0.45 μm MCA hydrophilic PTFE syringe filters. This method varies from the work reported by Abhishek [29], where liquids were separated using a centrifuge and filtered using a 0.22 μm filter. In theory, both gravity settling and centrifugal separation should only remove particle aggregates and rock particles and keep stable suspended particles unaffected. After the filtration, pH and UV–Vis spectrophotometric measurements followed.

#### 3.2.1. UV–Vis Spectrophotometry

Measurements were performed using a *Thermo Scientific Evolution 201* (Thermo Fisher Scientific, Waltham, MA, USA). It features a usable wavelength range of 190 to 1100 nm in combination with a quartz QX 10 mm cuvette. The fluid in the blank vail was the respective solvent of the NP solution: either TW, FW or AS. This method deviates from the methodology Abhishek [29] uses, as all measurements are compared to the absorbance in deionised water (DIW). This methodology was tested as well by comparing TW, FW and AS vs. DIW. The influence of the dissolved salts led to the decision to use each respective solvent instead of DIW as a reference. As a first step, various calibration concentrations were mixed for all three brines. Then, they were stored for 24 h and filtered using 0.45 μm MCA hydrophilic PTFE syringe filters, and their absorbance was measured. Additional measurements were conducted to investigate various influencing factors: (a) TW/FW against DIW; (b) 3000 ppm Na_2_CO_3_ in TW against TW; (c) 30 ppm tracer in brine against brine; (d) Impact of various filters; (e) Solution age (instant, 10 d, 14 d); (f) Impact of glass vs. plastic bottles at 22 °C and 60 °C; (g) Device drift over time: repeated measurements over time; (h) Repeatability of measurements.

#### 3.2.2. Limitations of Measurement—Exclusion Criterion

Some of the obtained nanofluid’s absorbance signals were too small compared to the baseline correction factors; hence, they were classified as noise. The decision was made to exclude measurements where the corrected absorbance was lower than 20% of the baseline values. The lower detection limit for the device in this configuration was found to be 0.005 wt%. Consequently, values below this threshold were excluded from the evaluation. This is attributed to the high influence of the rock and a corresponding high baseline combined with a low absorbance caused by the nanofluids. Therefore, the experimental set of samples was reduced to the ones further discussed in the result section. The formation of a filter cake in the syringe filters was assumed since it required considerable force to push the fluid through the filters compared to brine.

### 3.3. Single-Phase Core Flooding

**Figure 4:**
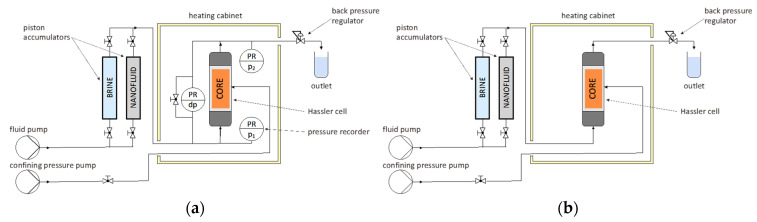
Core flooding setups used in this work for (**a**) permeability measurements and for (**b**) effluent analysis (nano single-phase floods) purposes. The core holder was placed vertically inside a heating cabinet, and in (**a**), pressure sensors were fitted to record the pressure differential across the core. In (**b**), fluid samples were collected after being injected into the rock at 60 °C. In both cases, a confining pressure of 35 bar was used.

Two types of core floods setup were used in this work and shown in Figure 4, one being used for permeability measurements (a) and the other for effluent analysis purposes (b). Brine and nanofluid were prepared and filled into piston accumulators. Core plugs were vacuum saturated in the respective brine for several hours and placed in a hassler cell. This was then mounted vertically inside a heating cabinet at 60 °C, and confining pressure of 35 bar was set. A backpressure regulator was set to 5 bar.

#### 3.3.1. Permeability Measurement and Nanofluids Injection

To measure the permeability to brine, various flow rates were injected to perform a step rate test, and the pressure response was recorded. Then, 60 mL of 0.1 wt% nanofluid in TW was injected into the core at 0.325 mL/min. The injection of nanofluid and brine at 0.325 mL/min correlates to an interstitial velocity of 0.046 cm/min (2.2 ft/day) and a Darcy velocity of 0.21 cm/min (10 ft/day). After the first injection, permeability to brine was again measured by conducting a step rate test.

Subsequently, the next injection was using 60 mL of 1 wt% nanofluid in TW at 0.325 mL/min and a subsequent step rate test. This showed the potential effect of nanomaterial treatment on permeability. Step rate tests with brine have been performed to evaluate the potential damage of nanofluid injection. Injecting brine before nanomaterials (baseline) and after nanomaterials allowed defining the possible damage. The permeability was assessed using Darcy’s equation. The injection of nanofluid and brine at 0.325 mL/min correlates to an interstitial velocity of 0.046 cm/min (2.2 ft/day) and a Darcy velocity of 0.21 cm/min (10 ft/day). After the first injection, permeability to brine was again measured by conducting a step rate test. The next injection was using 60 mL 1 wt% nanofluid in TW at 0.325 mL/min and a subsequent step rate test.

#### 3.3.2. Effluent Analysis Purposes

This setup did not contain pressure sensors but was extended by a sample collector after the backpressure regulator. Again, 60 °C and a vertical position of the Hassler cell were used. In a first step, TW was injected through the TW-saturated cores. Then, 60 mL of 0.1 wt% nanofluid in TW with a 30 ppm tracer was injected at 0.325 mL/min. After a sufficient volume of brine injection, 60 mL of 1 wt% nanofluid in TW with a 30 ppm tracer was injected, followed by a post-flush of brine. During all these steps, effluent samples of 3–6 mL were collected. These samples were either analysed via FFF, diluted with DIW by 1:3 for IC measurements or diluted with TW for UV–Vis spectrophotometry. The samples containing the 0.1 wt% nanofluid injections were diluted by 1:5 and samples with 1 wt% by 1:10.

UV–Vis Spectrophotometry measurements indicated contamination from the core by a peak at approximately 300 nm that led to the decision to dry the cores at 110 °C for several hours. Additionally, the core plugs were isolated from the rubber sleeve in the Hassler cell with aluminium foil. The pressure sensors used for permeability evaluation were removed because stagnant brine in the lines to the pressure sensors caused visible corrosion and were possible sources of contamination.

### 3.4. Effluents Analysis Methods

Four core floods have been conducted seeking to analyse effluents for nanoparticle and tracer concentration. Selected effluent samples were analysed using a Flow Field Flow Fractionation (FFF), Dynamic Light Scattering (DLS) and Multi-Angle Light Scattering (MALS). Each core flood comprised of (1) an initial flush with TW, (2) a low concentration nano injection, (3) TW flush, (4) a high concentration of nanofluid injection and (5) TW post flush.

To separate the solids by size in the sample prior to the measurement, effluent samples were fractionated using an AF2000 Flow FFF System (Postnova Analytics GmbH, Landsberg am Lech, Germany). Consequently, samples were analysed using a PN3621 Multi-Angle Light Scattering (MALS) and a PN3704 Dynamic Light Scattering (DLS) system (both from Postnova Analytics GmbH, Landsberg am Lech, Germany) to measure particle size, a nanoPartica SZ-100V2 Series (HORIBA Europe GmbH, Barleben, Germany) was used. Additionally, effects on particle size caused by the brine were investigated. Therefore, 0.1 wt% of each nanofluid was mixed with TW and FW and analysed using a Malvern Zetasizer Pro (Malvern Panalytical Ltd., Malvern, UK).

### 3.5. Scanning Electron Microscope (SEM)

Core plugs of Berea and Keuper were cut into 1 cm disks to facilitate evaluations. Disks were vacuum saturated for several hours with 1 wt% nanofluid of both types in either TW or FW. After vacuum saturation, disks were dried in a vacuum oven at 60 °C. The dried disks were broken to expose a rough untouched surface. After mounting the rock pieces on the sample holders, sides were covered with silver, and the top was sputtered with gold. This cover of a thin gold layer is necessary to be electrically conductive and avoid overcharging on the surface [29]. Subsequently, SEM imaging was conducted using a TESCAN Mira3 (TESCAN Orsay Holding a.s., Brno, Czech Republic). A pre-emptive test was conducted to evaluate how the crystallisation of salt from brine influenced the SEM imaging. Overcharging was not observed, and NaCl crystals were distinctive in shape and size. Therefore, they could be identified using the energy-dispersive X-ray (EDX) feature included in the SEM imaging system. Note that samples that have been treated with alkali solution were excluded from SEM imaging since they became very challenging to be measured. Note that a section of the middle of each rock used in the core flood experiment has been investigated. A detailed analysis of over 180 SEM images was comprised; however, this chapter focuses on outlining differences in adsorption behaviour, and therefore, only a selection is presented.

## 4. Results and Discussion

### 4.1. Batch Sorption

**Figure 5:**
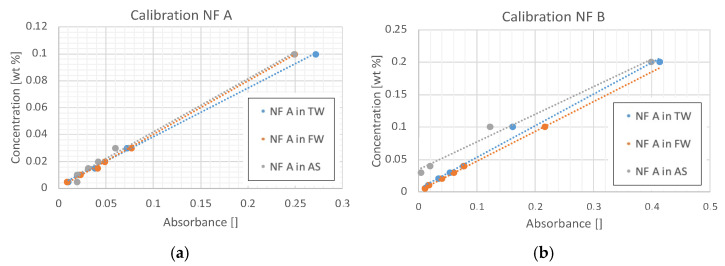
Calibration graph for different concentrations of NF A (**a**) and NF B (**b**) in TW, FW and AS. Solutions were left resting for 24 h after mixing and filtered using a 0.45 μm filter. A linear trend was found to fit the measured behaviour best for all solutions. These calibration plots enable the calculation of nanoparticle concentration from an absorbance measurement.

Various concentrations of nanofluid and brine were mixed, and their UV–Vis signal was measured to establish calibration plots. Plotting absorbance vs. concentration shows a linear relationship. The generated calibration constants that form the calibration plots shown in Figure 5 are listed in Table 4 They were used to calculate nanoparticle concentration from absorbance signals.

Blank samples consisted of the same mixture of brine and nanoparticle; however, they did not contain the respective rock material. To investigate the influence of rock and brine, reference samples were examined, which only contained brine and rock material. The absorbance signal of brine and rock without nanoparticles was taken as a baseline value, and correction factors *Abs_corr,i_* are shown in Table 4. These correction factors account for the influence each rock type has on the UV–Vis measurement in a certain brine and temperature when no nanofluid is present. Note that the calibration plots shown in Figure 5 enabled the calculation of the nanoparticle concentration from an absorbance measurement. For this case, a linear trend was found to fit the measured behaviour best for all nanomaterial solutions.

The measured values were evaluated using Equation (1) and the calibration coefficients and the correction factors stated in Table 4.

(1) $$c_{NF,\;i}=k_{i}\cdot \left(Abs-Abs_{corr}\right)+d_{i}$$

A reduction from the initial nanoparticle concentration to the calculated residual concentration *c_NF,i_* was consequently accounted for as nanoparticle adsorption to the rock material. Therefore, relative adsorption compared to the initial concentration (%) and absolute adsorption (wt%) was calculated.

#### 4.1.1. Nanofluid A

The results obtained for NF A are presented in Table 5. for Berea and Keuper. In Berea outcrops, adsorption was similar across the brines with values of 92% (FW), 91% (AS) and 88% (TW). The high adsorption values for AS could be explained by the relatively high baseline values for alkali. It was observed that temperature had a minor effect on the adsorption behaviour. Moreover, the addition of NF A did not alter the pH significantly in the investigated samples. Once the crushed rock was added to mixtures of NF A with brine, the pH was slightly reduced by 0.3 in TW and 0.35 in FW.

In Keuper outcrops, adsorption of NF A was similar in TW and FW with 77–80% at 22 °C and 87–88% at 60 °C. Here approximately 10% higher adsorption was seen in higher temperatures. Brine containing alkali showed the lowest adsorption (77%). The pH values were very similar across the samples, with pH 8.5 for TW, pH 7.1 for FW and pH 9.85 for AS.

#### 4.1.2. Nanofluid B

Table 6 shows the results for NF B in Berea and Keuper. In Berea outcrop samples, adsorption was 94% (FW), 86% (TW) and 61% (AS). Adsorption in alkali samples was significantly lower compared to samples in TW and FW. The effect of temperature is not significant in TW and FW; however, Berea samples showed high baseline values in TW at 60 °C. The addition of rock material to the nanofluid in FW increased the Ph from 4.71 to 6.36 (60 °C). Data obtained for 0.03 wt% solutions suggest that, at this concentration, nanofluid adsorption is high. Hence, the residual nanofluid concentration cannot be detected when the fluids contact the crushed rock.

For crushed Keuper material, nanoparticle adsorption was 93% (FW), 83% (TW) and 61% (AS). Adsorption in samples containing AS was significantly lower compared to the samples without. The effect of temperature seemed to be minor compared to the influence of the crushed rock material, which resulted in high baseline absorbance values. The addition of Keuper material to FW and NF B resulted in an increase from pH 4.71 to pH 6.36.

### 4.2. Core Flooding—Permeability Measurements

**Figure 6:**
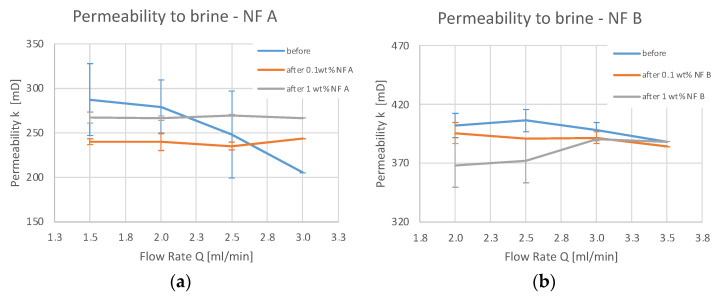
Permeability to brine was measured before a nanofluid injection (blue), after an injection of 0.1 wt% of NF (orange) and after an injection of 1 wt% NF (grey). NF A (**a**) and NF B (**b**) were both diluted in TW, and all permeability measurements were conducted with TW.

Permeability to brine is plotted in Figure 6**.** for each step of the injection sequence. Note that permeability to gas was ~490 mD for the tested Berea cores, whereas permeability to brine was considerably lower. This observation is in agreement with the work of Tanikawa and Shimamoto [30]. Permeability to brine (before and after) proved that injecting the tested nanofluids does not reduce the permeability to brine considerably. Therefore, Figure 6 depicts the permeability variation before and after nanomaterial injection, further addressed in the discussion section.

### 4.3. Core Flooding—Eflluents Analysis

For all plots in this section, nanofluid (NF) and tracer injection start at 0 pore volume (PV) on the abscissa, and the end is marked with a dashed vertical line.

#### 4.3.1. Nanofluid A in Berea

**Figure 7:**
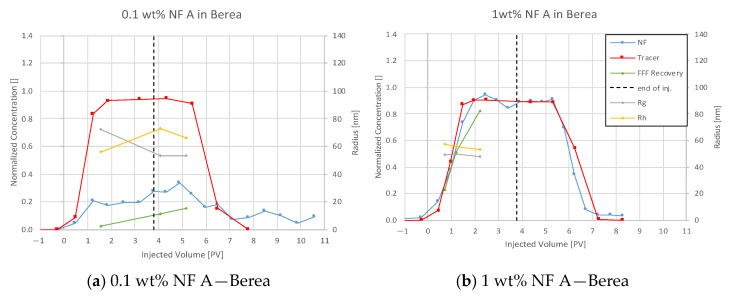
Effluent analysis for 0.1 wt% (**a**) and 1 wt% (**b**) NF A in TW in Berea sandstone. For the low concentration injections, high adsorption is seen for NF A, whereas the tracer seems to pass the core unaffected. During the high concentration injection of NF A, the effluent concentration follows the tracer concentration better, indicating no further adsorption. Particle size measurements confirm the low NF recovery (green) and larger particles arriving earlier in the effluent.

Effluent analysis for 0.1 wt% and 1 wt% NF A injections in Berea is shown in Figure 7. Plot (a) shows that the nanoparticle concentration in the effluent does not rise considerably during the first injection. The tracer included in the nanofluid injection was detected and had a distinctively shaped breakthrough curve. After a flush with TW, the second injection caused an almost parallel increase in both tracer and nanoparticle concentration. This behaviour might result from saturation with nanomaterials and no further adsorption in the core. It is worth noticing that both the tracer and the 1 wt% nanofluid injection reach a stable plateau at a lower concentration than the injection concentration. A comparison between tracer effluent history for both injection steps is presented in Figure 4, Figure 5 and Figure 6. The breakthrough is similar for injection steps, with 50% of the tracer concentration arriving after ~0.95 PV. After a plateau, the concentration reduces in the second injection step slower.

**Figure 8:**
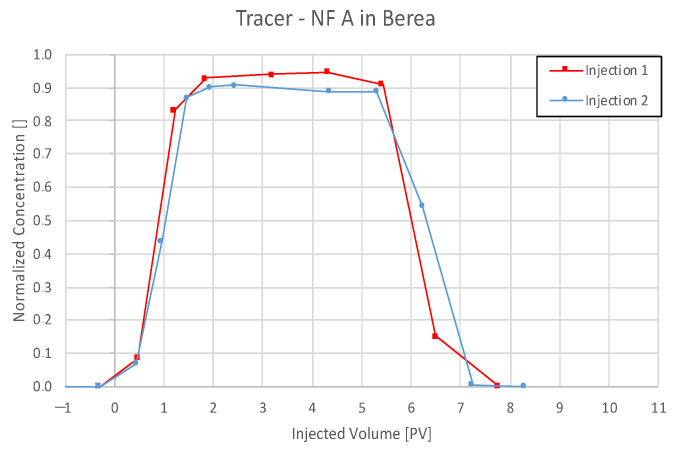
A comparison of tracer concentration history indicates a similar tracer breakthrough at ~0.95 PV (50%) for both injection steps and slightly longer retention in the second injection.

A nanoparticle concentration calculation via the DLS measurements shows a similar low recovery in the first injection step as the UV–Vis spectrophotometry. Hydrodynamic radius (Rh) describes the radius of the particle including a hull of solvent. Therefore, usually, Rh is slightly bigger compared to the radius of gyration (Rg). The results for the first measurement conducted in the first injection (a) shown in Figure 7 and Figure 8 indicate otherwise. This may be explained by the low concentration of recovered nanofluid resulting in an error of 4.3% (Rg) and 8.3% (Rh) compared to an average error of 2.5% (Rg) and 1.4% (Rh) in Keuper samples. The effluent history shows a slight decrease in size (second injection), indicating that bigger particles move faster through the core.

The obtained effluent histories were used to calculate the mass of produced nanofluid and tracer by integration, and the results are presented in Table 7. The ratio of produced over injected tracer is ~85%, indicating a baseline value for recovery of an inert chemical. The recovery of NF A in the second injection step is almost at that level (79.3%), whereas it is only 22.5% of the first injection. These results lead to a specific adsorption of 0.455 mg/g and 1.215 mg/g (nanoparticle/rock), respectively.

#### 4.3.2. Nanofluid A in Keuper

**Figure 9:**
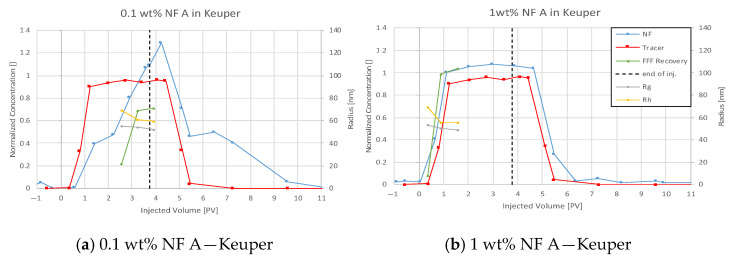
Effluent analysis for 0.1 wt% (**a**) and 1 wt% (**b**) NF A in TW in Keuper sandstone. A delayed breakthrough of nanomaterials can be seen in (**a**) peaking at a higher concentration than injected. An earlier nanoparticle breakthrough is seen in (**b**) compared to the tracer. Particle size measurements confirm UV–Vis concentration results and show larger particles arriving faster in the effluent.

The effluent history for NF A in Keuper shows a significant difference to Berea, as seen in Figure 9. The low concentration injection of NF A results in a delayed increase in nanoparticle concentration compared to the tracer. The NF concentration peaks at 0.13 wt% and decreases to zero with a delay compared to the tracer. The second injection shows NF concentration increase earlier or at least simultaneously to the tracer.

**Figure 10:**
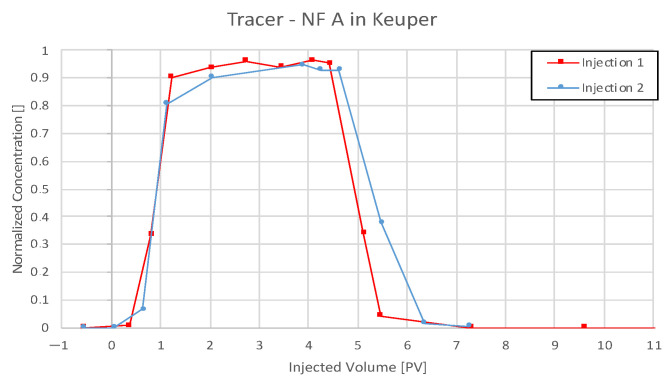
Tracer concentration history for Nanofluid A in Keuper in two injection steps. An identical tracer breakthrough can be observed at 0.9 PV (50%), whereas the plateau is reached slower. A delayed decrease in concentration for the second injection step can be observed.

The comparison between tracer concentration history for both injection steps seen in Figure 10 shows a parallel breakthrough of tracer. However, the plateau is reached slower, and a delay in concentration reduction can be observed for the second injection step. Particle size measurements reveal that larger particles elute earlier compared to smaller ones for both injection steps.

The mass balance calculated from these concentration profiles shows a tracer recovery of 85–90% (Table 7). Nanoparticle recovery of ~105% for UV–Vis measurements are confirmed by FFF recovery during the second injection. A calculation of negative specific adsorption is therefore meaningless since it can be assumed the core does not produce nanomaterials.

#### 4.3.3. Nanofluid B in Berea

**Figure 11:**
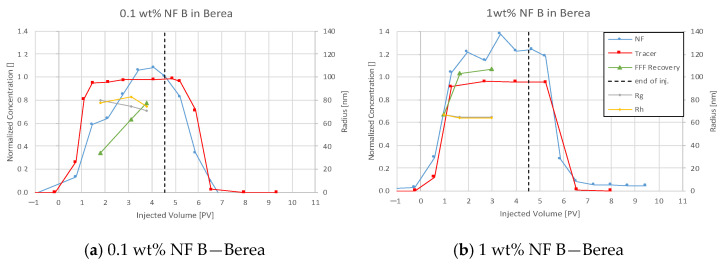
Effluent analysis for NF B in for 0.1 wt% (**a**) and 1 wt% (**b**) in TW in Berea sandstone. The calculated maximum concentration exceeds the injected concentration. The nanofluid shows only a slightly delayed breakthrough compared to the tracer. In the first injection step, the nanofluid shows a delayed breakthrough forming a peak at 0.11 wt%. Effluent concentration decreases faster compared to the tracer.

**Figure 12:**
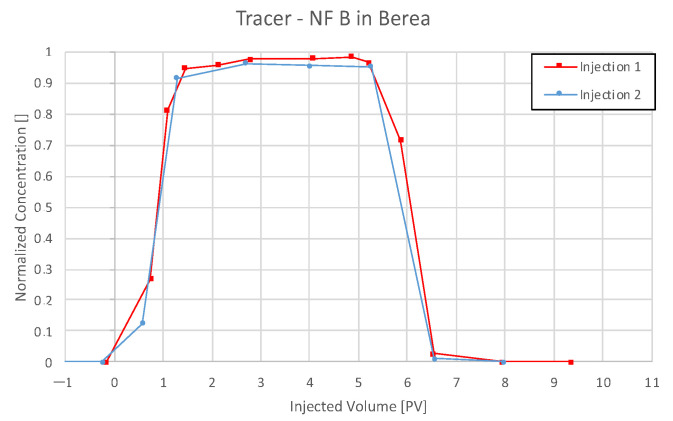
Tracer breakthrough comparison for NF B in Berea shows both breakthroughs at 0.9 PV. Particle size analysis suggests that larger particles arrive earlier in both injection steps.

Nanofluid (NF) B showed little delay to the tracer breakthrough in Berea, as seen in Figure 11. In the first injection step, the nanofluid shows a delayed breakthrough forming a peak at 0.11 wt%. The effluent concentration decreases faster compared to the tracer. In the second injection step, nanomaterials are now delayed to the tracer but also exceed the injected concentration. This behaviour creates a calculated NF recovery of ~77% and ~112%, respectively. FFF and DLS data confirm these concentration measurements by showing an NF concentration of 106% compared to blank samples. Particle size analysis suggests that larger particles arrive earlier in both injection steps. The comparison seen in Figure 12 shows a very similar trend for tracer concentration history in both injection steps. Mass balance calculation (Table 8) of the first injection step results in a specific adsorption of 0.09 mg/m^2^ and 0.13 mg/g (nanoparticle/rock). Effluent analysis for 0.1 wt% and 1 wt% NF.

#### 4.3.4. Nanofluid B in Keuper

**Figure 13:**
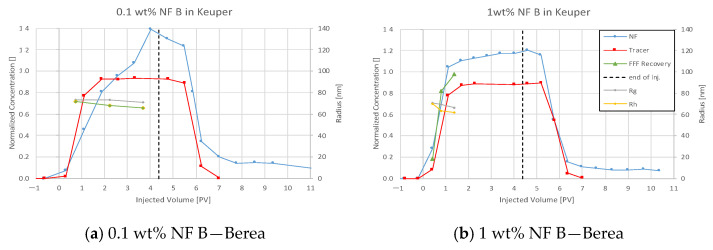
Effluent analysis for NF B in for 0.1 wt% (**a**) and 1 wt% (**b**) in TW in Keuper sandstone. Effluent analysis for nanofluid (NF) B in Keuper shows concentration peaks exceeding the injected concentration. In both cases, the calculated concentration remains at a constant level after the injection. The maximum measured NF concentration was 0.14 and 1.2 wt%, exceeding the respective injection concentration. The concentration does not reduce completely to zero after the injection and stays at ~12% in both cases.

Effluent analysis for the Keuper outcrop’s core is seen in Figure 13 for 0.1 wt% and 1 wt% NF B in Keuper. The maximum measured NF concentration was 0.14 and 1.2 wt%, exceeding the respective injection concentration. Furthermore, the concentration does not reduce completely to zero after the injection and stays at ~12% in both cases. These two factors suggest the elution of other UV light-absorbing material, which could result in an increased calculated concentration. For the first injection step, FFF and DLS data show a nanofluid concentration below the injected concentration. However, the values are considerably lower compared to results obtained via UV–Vis for all four core floods. Nanofluid breakthrough appears to be only slightly delayed for the first injection and earlier for the second injection. As particle size measurements indicated in all previous experiments, bigger particles are eluted faster than smaller ones.

**Figure 14:**
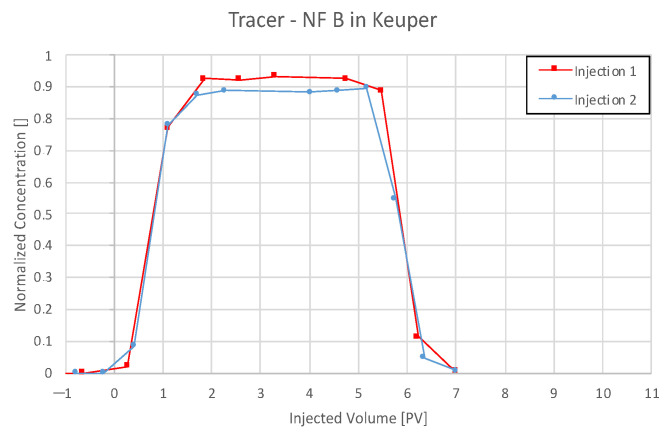
Tracer analysis for nanofluid (NF) B in the Keuper outcrop’s core. The concentration history is almost parallel for both injection steps.

Since the NF concentration did not reduce to zero, the mass balance calculation had to be adapted. The concentration was assumed to be zero after 7 PV, where tracer concentration was zero. Still, the calculated NF recovery exceeded 100%, as seen in Table 8. Tracer data comparison for nanofluid (NF) B in Keuper can be seen from Figure 14.

### 4.4. Flow Field Flow Fractionation (FFF) and Particle Size Measurements

Measurements comparing the effect of brine on particle size can be seen in Table 9. The results for the radius of gyration (Rg) and hydrodynamic radius (Rh) did not differ significantly across the used concentrations. A difference in size was not observed between samples diluted in FW and TW for both nanofluids. NF B showed higher values for hydrodynamic radius compared to the radius of gyration, whereas both values were very similar.

### 4.5. Scanning Electron Microscopy (SEM)

#### 4.5.1. Effect of Minerology

**Figure 15:**
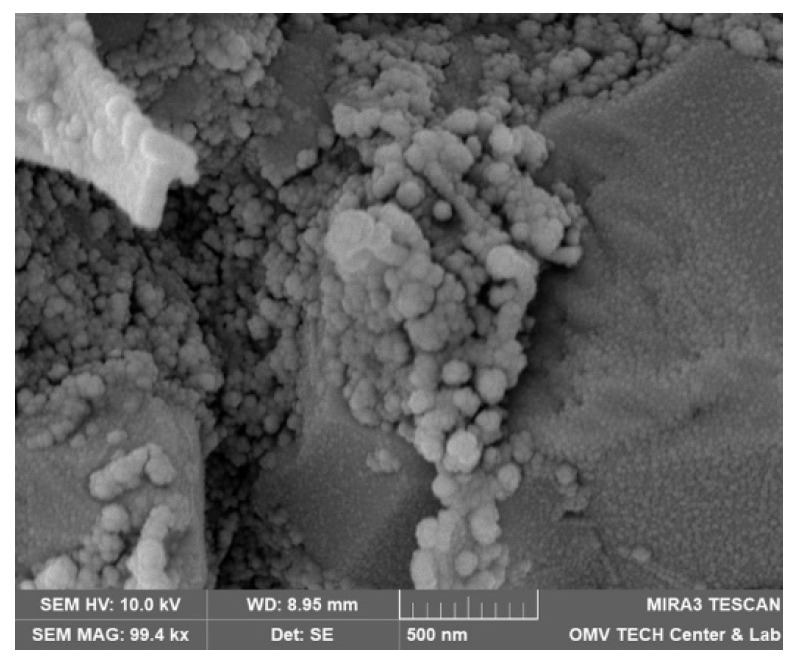
Scanning electron microscopy (SEM) image of a core flood rock sample following an injection of 1 wt% nanofluid A in test water.

**Figure 16:**
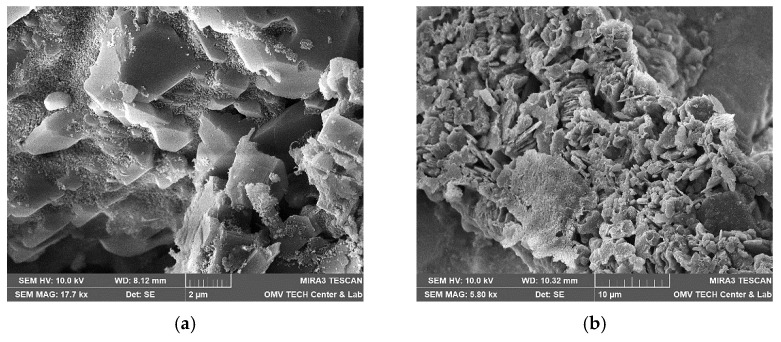
Scanning electron microscope (SEM) images measured for the core material used in this work. In (**a**), nanomaterials can be observed on almost all surfaces of minerals. The only exception is quartz cement with its distinctive smooth faces (Vacuum saturation of 1 wt% NF A in formation water; Berea). In (**b**), this overview shows various clay minerals that are coated completely with a layer of nanomaterials (Vacuum saturation of 1 wt% NF B in FW; Keuper).

**Figure 17:**
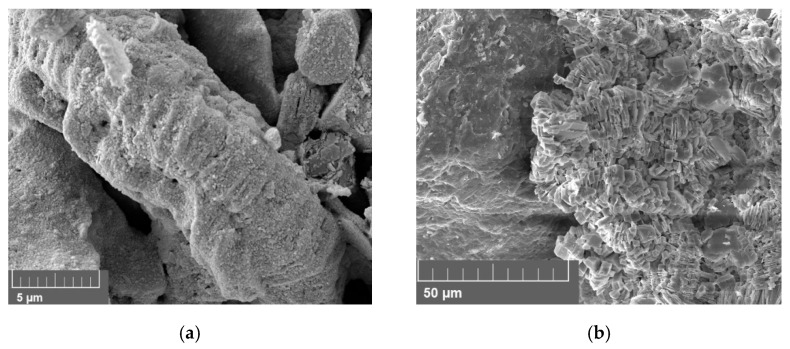
Scanning electron microscope (SEM) images measured for the core material used in this work. A comparison between nanoparticle-covered (**a**) and clean (**b**) kaolinite structures. Note that the smoothed edges in the left image are signs of weathering and not caused by the nanoparticle treatment ((**a**): core flood sample of 1 wt% NF A in test water, Keuper; (**b**): untreated sample, Berea).

Both nanofluids showed adsorption to all minerals present in the rock, regardless of the used brine. Figure 15, for instance, shows the formation of the nanoparticle clusters after injecting Nanofluid A dissolved in test water. Spots not completely covered in nanomaterials were usually smooth quartz cement faces, as seen in Figure 16a. However, the adsorption of nanomaterials in clusters was also observed on those. Clay minerals present in the rock such as kaolinite, chlorite, illite and iron oxide minerals were coated, as seen in Figure 16b and Figure 17.

**Figure 18:**
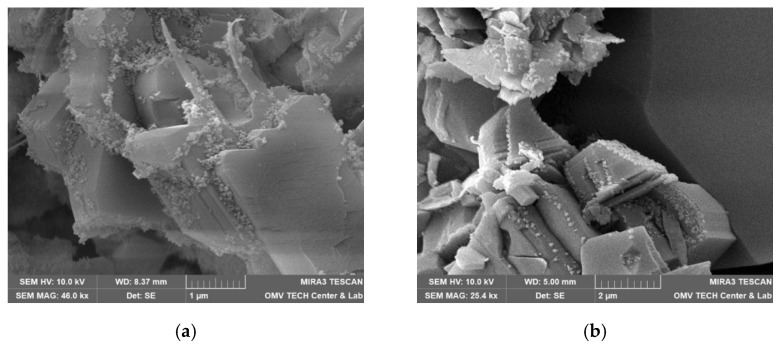
Scanning electron microscope (SEM) images measured for the core material used in this work. Nanomaterials are adsorbed in patterns parallel to mineral edges. The images show vacuum saturation samples of 1wt% NF A in FW (**a**) and 1wt% NF B in TW (**b**).

**Figure 19:**
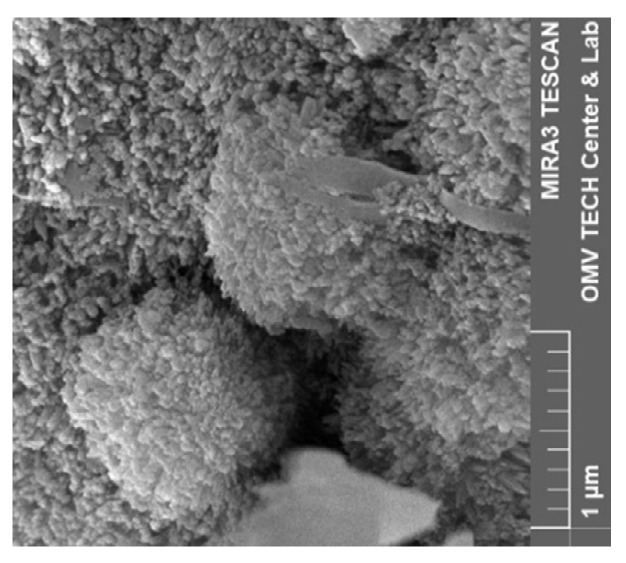
Scanning electron microscopy (SEM) image. Nanomaterials were also observed to adsorb on iron oxides, as seen in this image. The nanomaterials seem to form towers that are directed away from the centres of these mineral spheres. (Vacuum saturation of 1 wt% NF A in FW; Berea).

Interesting adsorption patterns can be seen in Figure 18, where nanomaterials are aligned with mineral edges in distinctive patterns. Figure 19 shows adsorption to spherical iron oxide minerals that seem to form towers radiating away from the spheres.

#### 4.5.2. Effect of Brine

**Figure 20:**
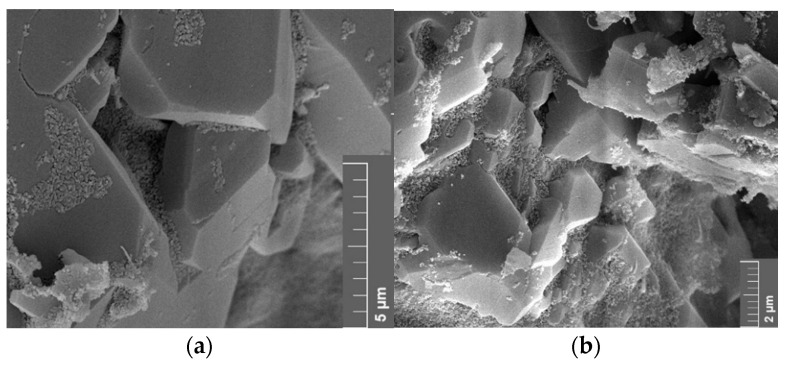
Scanning electron microscope (SEM) images measured for the core material used in this work. This image shows a comparison between adsorption of NF A to Berea in formation water (**a**) compared to TW (**b**). Surfaces in the FW sample were coated slightly more with nanomaterials. (left: Vacuum saturation of 1 wt% NF A in FW, Berea; right: vacuum saturation of 1 wt% NF A in TW, Berea).

**Figure 21:**
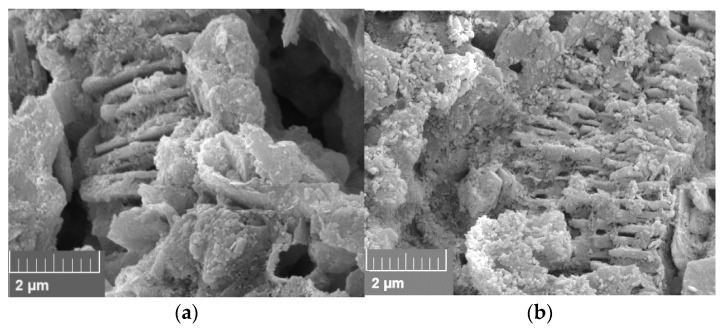
Scanning electron microscope (SEM) images measured for the core material used in this work. Comparison between NF B in formation water (**a**) and test water (**b**) on kaolinite structures found in Keuper rock. The sample treated with NF B in TW shows slightly higher adsorption of nanomaterials ((**a**): Vacuum saturation of 1 wt% NF B in FW, Keuper; (**b**): Vacuum saturation of 1 wt% NF B in TW, Keuper).

Vacuum saturated samples with nanofluid diluted in FW and TW showed slightly higher adsorption in FW. This can be seen in Figure 20 for NF A in Berea. In NF B, this effect was not as strong pronounced, as seen in Figure 21 in the kaolinite structures found in Keuper.

#### 4.5.3. Vacuum Saturation vs. Core Flood

**Figure 22:**
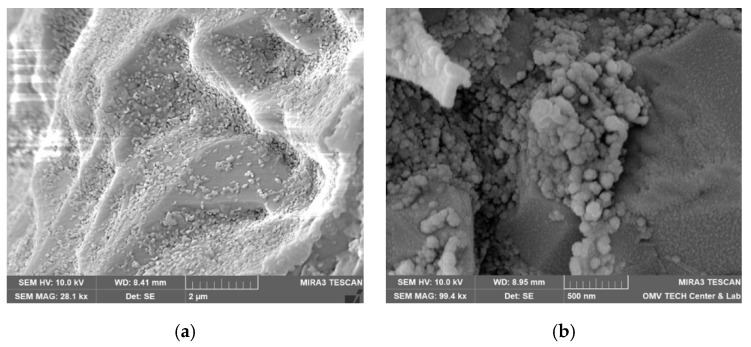
Scanning electron microscope (SEM) images measured for the core material used in this work. The image (**a**) shows a rock sample that has been vacuum saturated, whereas the image (**b**) shows a core flood sample. Both samples have been treated with 1 wt% nanofluid A in test water. Bigger particle structures can be seen on the core flood sample.

**Figure 23:**
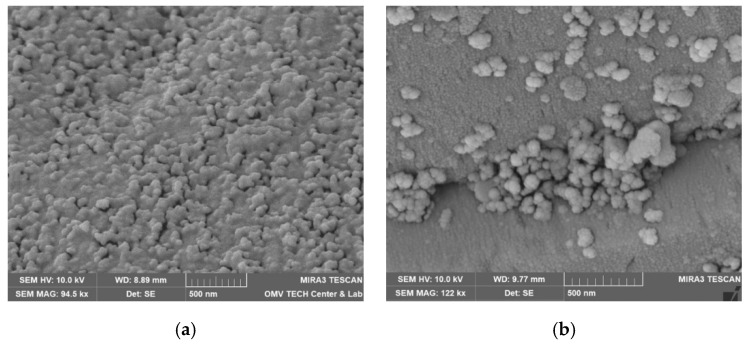
Scanning electron microscope (SEM) images measured for the core material used in this work. This comparison shows a rock sample that has been vacuum saturated (**a**) and a core flood sample (**b**). Both samples have been treated with 1 wt% nanofluid B in test water; however, bigger particle agglomeration can be seen in the core flood image.

SEM images of samples used in core floods show variations of the vacuum saturated samples. Direct comparisons for 1 wt% NF A and B in TW are seen in Figure 22 and Figure 23. Nanomaterials are adsorbed in bigger clusters in the core flood image compared to monolayer adsorption in the vacuum saturation sample.

## 5. Discussion

### 5.1. Discussion of Batch Sorption Results

The reduction in the nanoparticle concentration in batch adsorption can be attributed to two main reasons: particle aggregation (colloidal instability) or adsorption to the minerals. The formation of aggregates would result in filtration by the syringe filter (0.45 µm mesh). As seen in Figure 15, the particle structures formed during a core flood are almost 500 nm in size. If the agglomeration behaviour between batch sorption and core flood is similar, these structures would be filtered out by the syringe filter. Note that the formation of a filter cake was assumed since it required a higher force to filter batch sorption samples compared to other fluids. This is in agreement with Li et al. [31], who found a severe influence of nanomaterials of the same manufacturer on permeability as discussed in detail in Section 4.3.1.

The specific adsorption values are considerably higher compared to the ones observed in the core flood experiments. This could be explained by the higher fluid to rock ratio (4:1) used in batch sorption experiments compared to the core flood experiments (5:3). Despite similar results for the specific surface area, the crushed rock material could provide new binding sites for nanomaterials since the rock is freshly broken. The high adsorption in FW samples could be explained by the presence of divalent cations in the brine, which results in a higher ionic strength. An increased ionic strength compresses the electric double layer (EDL) and therefore weakens particle repulsion forces [32]. Hence, electrostatic stabilisation is expected to fail in these conditions since the energy barrier for particle agglomeration is lowered, and the kinetic energy increasingly dictates particle aggregation [33]. The compression of the EDL was also thought to be the primary influence of particle aggregation in the presence of NaCl in the work presented by Pham and Nguyen [34]. The observation of the highest absorption in FW and lowest in the alkali solution could also be explained with their respective pH. For unmodified silica nanomaterials, a higher pH results in a more negative Zeta potential and, therefore, higher particle repulsion. This effect could be less pronounced for the used nanomaterials since Zeta Titration plots (Figure 2) suggest similar Zeta potential values across the applied pH range. Additionally, nanomaterials could be in competition with weakly associated alkali cations, as described by Qiu et al. [35]. According to Van den pol et al. [36], alkali consumption is increasing with cation exchange capacity (CEC).

The work conducted by Pham and Nguyen [34] showed reduced adsorption in higher concentrations of nanofluid. They suggested surface modifications provide stability to the dispersion beyond a certain concentration threshold. The described trend is difficult to evaluate due to weak response in UV–Vis measurements for 0.03 wt% solutions. Li et al. [23] reported similar problems detecting UV–Vis signals at concentrations as low as 0.05 wt%. The observations made in the batch sorption experiments attribute temperature a minor effect in adsorption behaviour do not confirm the work presented by Pham and Nguyen [34]. There, an increased nanoparticle aggregation rate at elevated temperatures was attributed to the higher kinetic energy and more frequent particle collisions.

As previously mentioned, the impact of pore size distribution was not evaluated since it was regarded as not relevant. Instead, BET-specific surface area measurements were used as they provided more insight into the adsorption process. Since the cores used in this work are sandstone outcrops, they have fairly homogenous pores (8–16 µm). However, looking at adsorption from a pore size level, one has to understand that the used nanoparticles in a non-agglomerated form are orders of magnitude smaller than pore throats (Zhang et al. [22]). Therefore, a nanoparticle approaching the pore wall would not be influenced by the opposing pore wall. The prevailing type of adsorption seen later on in SEM images is in a single layer similar to the model described by Langmuir. This observation underlines the previously mentioned theory. Pore size distribution, however, has an impact on the specific surface area. A rock with a smaller average pore size with the same porosity has a higher specific surface area. This higher surface area results in more available spots for nanoparticles to be adsorbed.

### 5.2. Discussion of Core Flooding—Permeability Measurements

The formation of a filter cake was not visible, which is in contrast to the work of Bila and Torsæter [37], who investigated similar products to NF A in two phase experiments on Berea core plugs. There, the formation of a filter cake and higher displacement pressures were observed in oil displacement tests with crude oil. One could assume that the presence of oil enhanced the possible filter-cake formation as compared to single-phase evaluations here presented.

Various studies have been conducted investigating fines migration behaviour. If the ratio between particle to host diameter (d/D) is between 0.01 and 0.6, the particles can form bridges and block pores [38,39]. The formation of aggregates would be necessary to block pore throats since the nanomaterials themselves are orders of magnitude smaller than typical pore-throat diameters. Nanoparticle retention is mainly caused by physicochemical interaction with the porous media [22,40]. Since the tested nanofluids did not reduce the permeability to brine considerably (as seen from Figure 6), a formation of aggregates of sufficient size to block pores could be ruled out. Scanning electron microscopy (SEM) images discussed in detail in Section 4.4 support this observation, showing adsorbed particles on the rock. The formation of the nanoparticle clusters seen in Figure 15 is assumed to be insufficient to block pore throats and cause a reduction in permeability. This is in agreement with the work of Yu et al. [41], who studied the adsorption and transport of nanomaterials in porous media. They observed no effects on permeability in sandstone, whereas severe plugging occurred in dolomite and limestone samples. A detailed investigation on the effects of hydrophilic (FNP) and hydrophobic fumed silica nanomaterials (FNP-O) by *Evonik Operations GmbH* (Hanau, Germany) was conducted by Li et al. [23]. Their work provided evidence that 0.05 wt% FNP in 30 g/L NaCl reduced permeability to brine by a factor of 200.

### 5.3. Discussion of Core Flooding—Effluent Analysis Nanofluid A—Berea and Keuper

**Figure 24:**
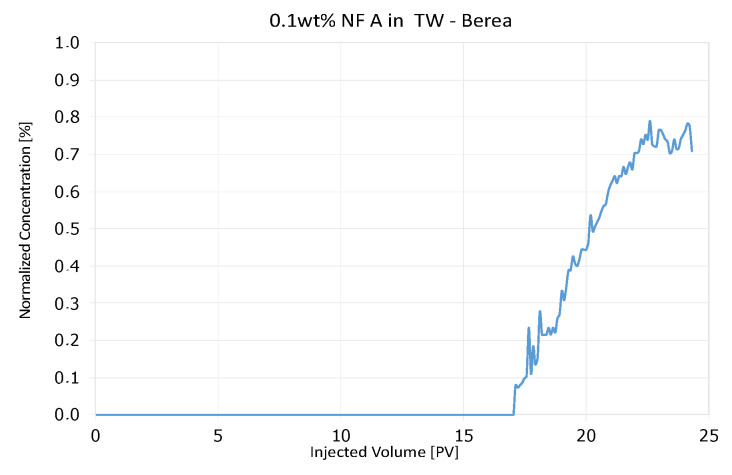
Preceding tracer test showing a nanofluid breakthrough after 17 PV. In this core flood, 0.1 wt% NF A in TW was injected and nanoparticle concentration was evaluated using DLS.

The adsorption of a considerable amount of nanofluid resulting in a delay in nanoparticle breakthrough seen in the Berea core flood is in agreement with experiments performed by an external provider (Figure 24). In these experiments, 0.1 wt% NF A in TW was injected into a Berea core with 3.5 cm diameter with 7.8 mL/min, and the effluent was analysed using DLS. The recorded nanofluid concentration seen in Figure 24. shows no breakthrough until 17 PV had been injected. Specific adsorption values presented in Table 5 comply with results provided by Zhang et al. [22] for PEG-coated silica nanomaterials in Boise sand packs.

The NF concentration history shown for Keuper in Figure 9 suggests reversible retention of nanomaterials in the core, resulting in sorption and desorption. The early breakthrough of nanomaterials observed in plot (b) might indicate that the core is saturated with nanomaterials, and no further ones can be adsorbed. A delayed breakthrough of nanomaterials compared to the tracer was also observed by Abhishek et al. [41] and Li et al. [23]. The latter used PEG-coated silica nanomaterials by the same manufacturer as used in this evaluation. The delayed breakthrough was explained by the adsorption/retention of nanomaterials on the rock surface. The delayed decrease in nanoparticle concentration was explained by the desorption of reversibly attached nanomaterials.

The nanoparticle recovery exceeding 100% could be caused by the elution of other UV light-absorbing material. This effect was minimised by covering the rock cores in aluminium foil to reduce contact with the core holder’s rubber sleeve. Additionally, they have been dried in the vacuum oven at 105 °C after Soxhlet extraction. Pre-emptive tests indicated that the solvents used for this cleaning procedure had a strong effect on UV–Vis measurements.

### 5.4. Discussion of Core Flooding—Effluent Analysis Nanofluid B—Berea and Keuper

On the one hand, Nanofluid B showed less adsorption compared to NF A in Berea. The nanofluid recovery exceeding 100% in the second injection suggests the elution of other UV light-absorbing material as mentioned above. Based on this result, it can be assumed that the calculated values for specific adsorption seen in Table 8 might be not representative.

On the other hand, Nanofluid B appeared to have lower adsorption in Keuper cores compared to NF A. Nevertheless, a higher nanofluid recovery was observed in Keuper rock compared to Berea. The early nanoparticle breakthrough compared to tracer (second injection) suggests low adsorption to the rock and faster elution compared to the tracer. A possible explanation for this effect may be a saturation of the core during the first injection step. In the second injection step, adsorption sites for nanomaterials would be occupied, leading to an early breakthrough. Since the tracer has not been adsorbed to the core in the first injection step, it would pass through it in the exact same pattern in the second injection, which can be seen in Figure 13.

### 5.5. Discussion of Flow Field Flow Fractionation (FFF) and Particle Size Measurements

The used nanomaterials have high fractal dimensions, as observed in the reported measurements. This could explain why the particle size measured by DLS (Rh) is smaller than one via Rg, especially for NF B in DIW. However, the value for Rg for NF B in DIW (96 nm) is significantly higher compared to all other Rg values (FW and TW). The differences seen here might be explained by the strong influence of the device and experimental setup for particle size estimation. Values for nanoparticle solutions in DIW have been measured by the manufacturer, whereas all other measurements were conducted in the means of this work.

### 5.6. Discussion of Scanning Electron Microscopy

The results suggest that the mineral types present in the rock have a minor effect on the adsorption behaviour. A higher adsorption affinity for quartz over kaolinite, as described by Abhishek and Hamouda [26], has not been observed in these experiments. The effect of brine on adsorption behaviour seen in previous experiments was confirmed by these microscopy images. Higher nanoparticle adsorption was observed on FW samples for both rock types.

Adsorption in vacuum saturation samples was in single layers, whereas clusters have been observed in core flood samples. This suggests that the flow through the porous rock exerts a higher hydrodynamic force on the nanomaterials, as described by Zhang et al. (2015). Particles are pushed closer to each other or rock surfaces, which increases the influence of attractive vdW forces. The formation of a nanoparticle monolayer is different from the work of Abhishek and Hamouda [26], who observed adsorption in successive layers due to drying effects. The rock samples were stored in brine after vacuum saturation to wash off excess nanomaterials appeared to be a successful method to reduce these drying effects.

## 6. Conclusions

A study on possible formation damage and sorption of nanomaterials was conducted by cross analysing various laboratory data sources. The focus was given to evaluating fluid–fluid and fluid–rock interactions by means of compatibility tests, batch sorption experiments and core floods.

Berea and Keuper outcrop rock materials have similar porosities and specific surface areas. Permeability and clay content are higher in Keuper, although it has a higher degree of inhomogeneity. Zeta potential for both nanofluids indicates that the dispersion stability is provided by their surface modifications. Berea rock showed more potential for adsorption than Keuper rock in all conducted experiments.

The results suggest that formation water promoted the adsorption of both types of nanomaterials compared to test water due to the presence of divalent cations. The influence of pH on adsorption behaviour can be seen in the highest adsorption values seen in this brine and the lowest in alkali solutions. The temperature had a minor effect on nanoparticle adsorption behaviour. Surface roughness seems to be the dominant driving factor for adsorption site selection. Small nanoparticle clusters have been observed on core flood samples, whereas vacuum saturation samples showed adsorption in a single layer.

Core flood experiments showed that the injected nanofluids did not have a sufficient effect on permeability to be accounted for. A formation of a filter cake was not observed in the core flood experiments. A delayed nanofluid breakthrough compared to tracer suggests adsorption and saturation with nanomaterials. This leads to adsorption spots being occupied and an earlier elution compared to the tracer for a succeeding injection. It was observed that bigger nanomaterials move faster through the core.

## Figures and Tables

**Table 1:** Overall core and saturation data for the outcrop samples used in this work.

Parameter	Units	Berea ^1^		Keuper ^2^	
		Mean	SD	Mean	SD
Length	cm	6.95	0.02	8.12	0.09
Diameter		2.97	0.01	2.98	0.01
Grain Volume	kg/cm^3^	37.01	0.31	42.98	0.67
Porosity	%	21.92	0.121	23.54	0.794
*N*_2_ permeability (*k_g_*)	mD	485.00	32.00	1424.00	172.00
Water (Test Water) permeability (*k_w_*)		314.00	66.00	890.00	193.90
BET ^3^—Core Plug 60 °C	(m^2^/g)	1.4364	0.0051	0.9896	0.0028
BET ^3^—Core Plug 110 °C		1.6184	0.0064	-	-
BET ^3^—Crushed Cores 60 °C		1.5621	0.0032	1.5645	0.0055

^1^ Data from 10 core plugs. ^2^ Data from 8 core plugs. ^3^ Specific Surface Area (BET).

**Table 2:** Composition of synthetic brines used in this work.

Formulation	TW (g/L) Softened Injection Brine	FW (g/L) Synthetic Formation Brine
NaCl (g/L)	18.96	19.75
NaHCO_3_ (g/L)	1.85	-
CaCl_2_ ·2H_2_O (g/L)	-	0.400
MgCl_2_ ·6 H_2_O (g/L)	-	0.660
NH_4_Cl (g/L)	-	0.170
pH (23 °C)	8.46	6.53
Ionic Strength (M)	0.346	0.373

**Table 3:** Properties of Nanofluids (NF) used in this work.

Property	Nanofluid A	Nanofluid B
Density at 25 °C (g/cm^3^)	1.15 ± 8 × 10^−4^	1.14 ± 1 × 10^−3^
Density at 60 °C (g/cm^3^)	1.13 ± 2 × 10^−3^	1.11 ± 4 × 10^−4^
Solid content (%) (loss on drying at 105 °C)	24.9	27.8
Viscosity at 10 1/s (mPa.s)	19	48
Viscosity at 100 1/s (mPa.s)	18	37
Particle size (d50) DLS (nm) ^1^	128	140
Particle size (d50) SLS (nm) ^1^	111	117
R_g_ (nm) ^2^	60 ^3^	96 ^4^
R_hyd_ (nm) ^5^	52	61
pH at 22 °C ^6^	8.99	2.82
pH at 22 °C ^6^ for 0.1 wt% in TW	8.53	8.49
pH at 22 °C ^6^ for 0.03 wt% in TW	8.56	8.55
pH at 22 °C ^6^ for 0.1 wt% in FW	7.14	4.91
pH at 22 °C ^6^ for 0.03 wt% in FW	6.96	6.16
pH at 22 °C ^6^ for 0.03 wt% in AS	9.90	9.87

^1^ measured in DIW; ^2^ Mean radius of gyration: online MALS, random coil model; ^3^ Particle size distribution 19–131 nm; D50 91 nm; ^4^ Particle size distribution 19–199 nm; ^5^ mean hydrodynamic radius; ^6^ measured in triplicates, SD 0.002.

**Table 4:** Calibration constants for 24 h aged nanofluid solutions after filtration at 270 nm and Absorbance correction factors for rock materials. These constants were later used to calculate nanoparticle concentrations from UV–Vis absorption measurements (FW: formation water, TW: test water, AS: alkali solution). Absorbance in different brines at two temperatures using doublet measurements and an average standard deviation for absorbance at 270 nm of 3.8 × 10^−3^.

Material	Brine	*k_i_*	*d_i_*	*R* ^2^	*Abs_corr,i_*	
					22 °C	60 °C
Nanofluid A	TW	0.3624	0.0020	0.9992	-	-
	FW	0.3984	0.0002	0.9991	-	-
	AS	0.3962	0.0023	0.9931	-	-
Nanofluid B	TW	0.4871	0.0046	0.9880	-	-
	FW	0.4579	0.0020	0.9982	-	-
	AS	0.4248	0.0346	0.9868	-	-
Berea	TW	-	-	-	0.031	0.047
	FW	-	-	-	0.019	0.027
	AS	-	-	-	0.048	0.091
Keuper	TW	-	-	-	0.054	0.133
	FW	-	-	-	0.023	0.044
	AS	-	-	-	0.088	0.237

**Table 5:** Adsorption results and pH measurements for NF A in Berea and Keuper. Absorbance measurements were performed in doublets with an average standard deviation of 1.8 × 10^−3^ for all fluids. pH standard deviation was defined as 2 × 10^−2^ for all fluids in average (FW: formation water, TW: test water, AS: alkali solution).

Core	Initial Conc. (wt%)	Brine	T	Residual Conc. *c_NF,i_*	Adsorption		Specific Adsorption		pH		
			(°C)	(wt%)	(wt%)	(%)	(mg/g)	(mg/m^2^)	B-R ^1^	NF-B-R ^2^	NF-B ^3^
Berea	0.1	TW	22	0.0118	0.0882	88	3.53	2.26	8.29	8.26	8.45
			60	0.0107	0.0893	89	3.57	2.39	8.24	8.29	8.58
		FW	22	0.0083	0.0917	92	3.67	2.35	6.77	6.75	6.94
			60	0.0097	0.0903	90	3.61	2.31	6.87	6.78	7.14
		AS	22	0.0088	0.0912	91	3.65	2.34	9.82	9.88	9.89
	0.03	TW	22	0.0102	0.0198	66	0.79	0.51	8.29	8.30	8.41
			60	0.0070	0.0230	77	0.96	0.61	8.24	8.37	8.63
		FW	22	0.0050	0.0250	83	1.00	0.64	6.77	6.79	6.80
			60	0.0050	0.0250	83	1.00	0.64	6.87	6.73	6.99
Keuper	0.1	TW	22	0.0227	0.0773	77	3.09	1.98	8.40	8.40	8.45
			60	0.0133	0.0867	87	3.57	2.29	8.49	8.45	8.58
		FW	22	0.0198	0.0802	80	3.21	2.05	7.03	7.15	6.94
			60	0.0119	0.0881	88	3.52	2.25	7.18	7.07	7.14
		AS	22	0.0233	0.0767	77	3.07	1.96	9.85	9.88	9.89
	0.03	TW	22	0.0116	0.0184	61	0.74	0.47	8.42	8.38	8.41
		FW	22	0.0070	0.0230	77	0.92	0.59	7.03	7.11	6.80
			60	0.0073	0.0227	76	0.91	0.58	7.18	7.15	6.99

^1^ B-R = Brine Rock; ^2^ NF-B-R = Nanofluid Brine Rock; ^3^ NF-B = Nanofluid Brine.

**Table 6:** Adsorption results and pH measurements for NF B in Berea and Keuper. Absorbance measurements were performed in doublets with an average standard deviation of 7.4 × 10^−4^ for all fluids. pH standard deviation was defined as 2 × 10^−2^ for all fluids in average. Note the increase in pH from 4.71 to 6.36 for 0.1 wt% NF B in FW at 60 °C. Note the increase in pH from 4.71 to 6.36 for 0.1 wt% NF B in FW at 60 °C. (FW: formation water, TW: test water, AS: alkali solution).

Core	Initial Conc. (wt%)	Brine	T	Residual Conc. *c_NF,i_*	Adsorption		Specific Adsorption		pH		
			(°C)	(wt%)	(wt%)	(%)	(mg/g)	(mg/m^2^)	B-R ^1^	NF-B-R ^2^	NF-B ^3^
Berea	0.1	TW	22	0.0125	0.0875	88	3.50	2.27	8.29	8.22	8.44
			60	0.0146	0.0854	85	3.42	2.19	8.24	8.27	8.61
		FW	22	0.0057	0.0943	94	3.77	2.41	6.77	6.21	4.96
			60	0.0068	0.0932	93	3.73	2.39	6.87	6.36	4.71
		AS	22	0.0387	0.0613	61	2.45	1.57	9.82	9.83	9.86
	0.03	FW	22	0.0051	0.0249	83	1.00	0.64	6.77	6.53	6.24
			60	0.0125	0.0243	81	1.01	0.65	-	6.74	-
Keuper	0.1	TW	22	0.0172	0.0828	83	3.31	2.12	8.40	8.30	8.44
		FW	22	0.0065	0.0935	93	3.74	2.39	7.03	6.64	4.96
			60	0.0066	0.0934	93	3.74	2.39	7.18	6.73	4.71
		AS	22	0.0468	0.0532	53	2.13	1.36	-	9.86	-
		FW	22	0.0062	0.0238	79	0.95	0.61	7.03	6.89	6.24

^1^ B-R = Brine Rock; ^2^ NF-B-R = Nanofluid Brine Rock; ^3^ NF-B = Nanofluid Brine.

**Table 7:** Adsorption results following mass balance calculation for NF A in Berea and Keuper.

Material	0.1 wt% NF A in TW		1 wt% NF A in TW	
	Berea	Keuper	Berea	Keuper
NF Recovery (%)	22.46	104.20	79.31	104.98
NF Adsorption (mg/m^2^)	0.317	-	0.846	-
NF Adsorption (mg/g)	0.455	-	1.215	-
Tracer Recovery (%)	85.49	84.72	84.37	90.05

**Table 8:** Adsorption calculation via mass balance following concentration calculation using UV–Vis spectroscopy data for nanofluid B in Berea and Keuper outcrop core.

Material	0.1 wt% NF A in TW		1 wt% NF A in TW	
	Berea	Keuper	Berea	Keuper
NF Recovery (%)	77.21	108.10	112.65	113.10
NF Adsorption (mg/m^2^)	0.090	-	-	-
NF Adsorption (mg/g)	0.130	-	-	-
Tracer Recovery (%)	90.35	89.85	89.50	85.87

**Table 9:** Particle size measurements for different concentrations of nanofluids (NF) in two brines (formation water (FW) and test water (TW). Rg: Radius of gyration (R50; MALS), Rh: Hydrodynamic radius (DLS). The values in deionised water (DIW) have been provided by Evonik Resource Efficiency GmbH.

Material	Brine	Concent. (wt%)	Rg (nm)	Rh (nm)
Nanofluid A	TW	0.1	48 ± 1.6%	56 ± 2.7%
		1	48 ± 0.5%	54 ± 1.4%
	FW	0.1	49 ± 0.1%	54 ± 0.5%
	DIW	-	60	52
Nanofluid B	TW	0.1	64 ± 0.8%	62 ± 1.6%
		1	66 ± 0.4%	67 ± 3%
	FW	0.1	68 ± 1.6%	64 ± 0.8%
	DIW	-	96	61

## Data Availability

Data presented in this article are available on request from the corresponding author.
